# Neurometabolic Evidence Supporting the Hypothesis of Increased Incidence of Type 3 Diabetes Mellitus in the 21st Century

**DOI:** 10.1155/2019/1435276

**Published:** 2019-07-21

**Authors:** Anna Rorbach-Dolata, Agnieszka Piwowar

**Affiliations:** Department of Toxicology, Faculty of Pharmacy with the Division of Laboratory Diagnostics, Wroclaw Medical University, Borowska 211, 50-552 Wroclaw, Poland

## Abstract

The most recent evidence supports the existence of a link between type 2 diabetes (T2DM) and Alzheimer's Disease (AD), described by the new term: type 3 diabetes (T3D). The increasing incidence of T2DM in the 21st century and accompanying reports on the higher risk of AD in diabetic patients prompts the search for pathways linking glycemia disturbances and neurodegeneration. It is suggested that hyperglycemia may lead to glutamate-induced excitotoxicity, a pathological process resulting from excessive depolarization of membrane and uncontrolled calcium ion influx into neuronal cells. On the other hand, it has been confirmed that peripheral insulin resistance triggers insulin resistance in the brain, which may consequently contribute to AD by amyloid beta accumulation, tau phosphorylation, oxidative stress, advanced glycation end products, and apoptosis. Some literature sources suggest significant amylin involvement in additional amyloid formation in the central nervous system, especially under hyperamylinemic conditions. It is particularly important to provide early diagnostics in people with metabolic disturbances, especially including fasting insulin and HOMA-IR, which are necessary to reveal insulin resistance. The present review reveals the most recent and important evidence associated with the phenomenon of T3D and discusses the potential lacks of prevention and diagnostics for diabetes which might result in neurometabolic disorders, from a pharmacotherapy perspective.

## 1. Introduction

The most recent reports indicate the existence of a link between diabetes mellitus (DM) and Alzheimer's Disease (AD), and it is suggested that people with diabetes manifest greater susceptibility to AD [1–4]. In 2013, Gudala et al. [3] published a meta-analysis of DM cases and the risk of all types of dementia (including AD) based on 28 prospective observational studies conducted on a total of 89708 patients with diabetes mellitus in comparison to a nondiabetic control group [3]. The results demonstrated that diabetic patients carried over a 70% risk of development of all types of dementia and almost a 60% risk of developing AD. Interestingly, recent data from the Centers for Disease Control and Prevention estimate that over 30.3 million Americans are living with diabetes including 30.2 million of people aged 18 or older, which is approximately 12% of U.S. population [5]. Furthermore, 1.4 million Americans are diagnosed with diabetes every year (American Diabetes Association) [6]. The data from 2018 indicated that the prevalence of diabetes in Europe had reached 10.3% for men, 9.6% for women, and the total number of diabetic patients oscillated around 60 million. In 2017 over 477.715 deaths were the consequence of diabetes [7]. Based on the International Federation of Diabetes (IDF) Atlas for Diabetes for 2013, the Middle East and North Africa (MENA) demonstrated a diabetes prevalence of approximately 10.9% [8]. In response to the dramatic increase in the number of diabetic patients, the United Nations approved DM as an epidemic of the 21st century (the only one noninfectious disease). As regards to AD, surprisingly, more than 100 years after the first case was confirmed, there is still no treatment available to prevent or inhibit the neurodegeneration progress; however, some drugs are used to treat the symptoms of AD which measurably improve quality of life. From 2002 to 2012, 244 drugs for Alzheimer's Disease were tested; nevertheless, only one of them received approval from the U.S. Food and Drugs Federation (FDA). According to the most recent data on Alzheimer's Disease, published in 2018, it is estimated that roughly 5.7 million Americans are diagnosed with AD, including approximately 5.5 million people aged 65 or over and about 200 000 with younger-onset Alzheimer's. Interestingly enough, these 5.5 million Americans included 3.4 million women and 2.0 million men, suggesting that women are more susceptible to AD [9]. In 2013, Hebert et al. [10] evaluated statistical AD prevalence for people aged 65 and over in the United States, from 2010 to 2050, based on the U.S. census. The results predicted a rapid increase in AD cases, up to 13.8 in the next 34 years unless prevention is developed. To this number must be added the number of potential diabetics who also become patients with AD. Whereas there is still no convincing explanation for the phenomenon of AD connecting with DM, it has resulted in some hypotheses focused on impaired insulin signaling, insulin–resistance in the brain [11] and hyperglycemia-induced excitotoxicity [12]. It is particularly important to realize that type 2 diabetes (T2D) is connected with insulin resistance, which cannot be diagnosed without a fasting insulin test and a HOMA-IR assessment, which is still uncommon, and not included in the worldwide diabetes guidelines. These considerations gave rise to the new term: type 3 diabetes (T3D) [13]. Herein, we focus on the unusual phenomenon of T3D, investigating diverse pathways of development, as this phenomenon is still mysterious.

## 2. Glutamate-Induced Excitotoxicity in Neurodegeneration

An important, but still little known, aspect of the development of neurodegenerative diseases is glutamate excitotoxicity, described as a state of excessive glutamate concentration which may lead to neuronal degeneration and consequently to neuronal death [14]. It has been confirmed that excitatory amino acids (EAAs)-neurotransmitters of the central nervous system (CNS), particularly glutamate, are the main agents responsible for neuronal death through the excitotoxicity effect [15]. Physiologically, glutamate is responsible for elementary perception and cognition in the brain, through the initiatory role of an excitatory response. The excitatory response occurs due to the interaction of glutamate with synaptic receptors connected with ion channels. Superabundant activation of these receptors, resulting in high levels of electrical and energetic activity oriented towards neurons, which may lead to neuronal degeneration, is called, precisely, excitotoxicity [16]. This glutamate excitatory effect may occur through the activation of the major ionotropic and metabotropic receptors. The three major ionotropic receptors are N-methyl-D-aspartic acid (NMDA), *α*-amino-3-hydroxy-5-methylisoxazole-4-propionate (AMPA), and kainic acid (KA) receptors [17]. Chronic, mild activation of NMDA results in pathological intracellular calcium overload and excitotoxicity [18]. Activation of AMPA receptors leads to an influx of Na+, Cl− ions and water, as well as a small amount of Ca2+ via receptor-gated channels which results in swelling of the neurons [19]. Recent studies suggest that overstimulation of non-NMDA receptors with KA may lead to autophagy and activation of lysosomal pathways, both responsible for excitotoxic neurodegeneration [20, 21]. The mitochondria ability to accumulate excessive amounts of Ca2+ in situ plays a crucial role in the excitotoxicity process. It has been confirmed that excessive influx of Ca2+ via NMDARs targets mitochondria resulting in Ca2+ overload, which consequently leads to mitochondrial dysfunction [22]. Increased intracellular Ca2+ level is responsible for the activation of Ca2+-sensitive protein kinases and the hyperphosphorylation of cytoskeletal proteins, especially tau protein and ubiquitin. Hyperphosphorylated tau and ubiquitin form part of neurofibrillary tangles. If the influx of Ca+ is permanent, the conditions trigger persistent depolarization of the neuronal cell membrane, which releases the Mg2+ block in the NMDA receptor leading to an increase of intracellular Ca2+. This excessive activation may result in both cortical and subcortical necrosis [23]. This is particularly important in the context of the relationship between hyperkalemia and brain fluid calcium concentration, which may suggest that attention should be paid to the contribution of parathormone and calcitonin to the regulation of calcium levels in patients with diabetes.

## 3. Theories of Hyperglycemia-Induced Neurodegeneration Leading to T3D

Chronic hyperglycemia, the integral circumstance of DM (both types: T1D and T2D), is believed to be a risk factor for AD [24] and result in T3D. Interestingly, type 3 diabetes is classified as a neurometabolic disorder, directly related to long-lasting metabolic disturbances accompanying T2D connected with hyperinsulinemia and insulin resistance, leading to full-blown Alzheimer's Disease [4, 25]. However, regarding the increased incidence of AD in T1D and T2D it is suggested that AD may be induced by different mechanisms in each condition. Kim et al. [26] carried out an experiment involving streptozotocin (STZ)-injected mice with induced type 1 diabetes and the commonly known db/db mice, a genetic model of T2D. The research hypothesis included the relationship between hyperphosphorylation, cleavage of tau, and diabetic susceptibility to AD [27]. The results proved that tau phosphorylation was increased in both db/db mice and (STZ)-injected mice; however the db/db mice model demonstrated more severely increased tau phosphorylation and cleavage in comparison to type (STZ)-injected animals. For the first time it was demonstrated that increased tau phosphorylation in a type 2 diabetic mouse brain connects with enhanced tau cleavage, suggesting that not only hyperphosphorylation but also the cleavage of tau seems to be significant factors in the development of AD. The major difference suggested was that in type 1 diabetes, insulin deficiency seems to be the main factor for increased tau phosphorylation, while in type 2 diabetes, hyperglycemia-induced tau cleavage with contributing insulin disturbances may lead to major tau pathology. Additionally, patients with T2D and AD demonstrate similar amyloid-*β* deposits both in the pancreas and in the brain [28].

It is also suggested that hyperglycemia conditions act by increasing lactate and hydrogen ion concentration and exaggerate the reduction of intracellular pH (pHi) and extracellular pH (pHe) [29]. Previously, Li et al. [30] reported the critical values for plasma glucose concentration in brain damage, including pHe measurements. They confirmed that, in values of 4-6 mM, damage only in the Sommer's Sector (the CA1 sector in the hippocampus) was observed. At glucose concentrations of 8-12 mM, moderate damage was confirmed rarely in the caudoputamen, the parietal cortex, and the thalamus. However, at glucose values above 12 mM, damage in these areas increased critically, and destruction also took place in additional structures (the cingulate cortex, the CA3 sector of the hippocampus, and the substantia nigra). The values of pHe measured in the parietal cortex demonstrated a limit for seizure induction in the range of 6.4-6.5, related to intracellular values, which are about 6.2-6.3.

**Figure 1:**
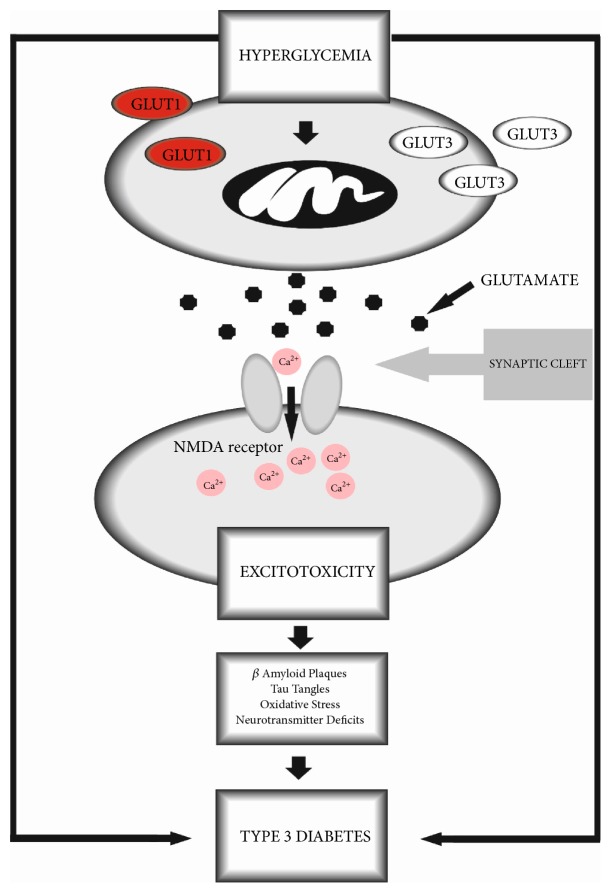
Proposed pathway of hyperglycemia-induced excitotoxicity leading to T3D.

The most interesting theories about chronic hyperglycemia and glutamate-induced excitotoxicity are still being analyzed. Some authors suggest that A*β* formation is associated with diabetic conditions of reactive oxygen and nitrogen species (respectively: ROS and RNS) production, which result in calcium-dependent excitotoxicity, disability of cellular respiration, and modification of synaptic functions, which consequently lead to learning and memory impairments [31]. Other scientists suggest that the role of hyperglycemia in AD pathogenesis is based on a modulating process of extracellular A*β* formation [32]. The authors propose the hypothesis of hyperglycemia-induced interstitial fluid (ISF) A*β* concentrations and modification of neuronal activity, leading to an increase in A*β* production. They explain that increased blood glucose level results in elevations in intracellular ATP, which triggers the closing of ATP-sensitive potassium (KATP) channels, and consequently leads to membrane depolarization and an increase in cellular excitability. Moreover, it results in increased neuronal activity and extracellular concentrations of A*β*. The authors suggest that this provides a potential therapeutic aim for AD; however, more studies are needed to explain the pathomechanism of hyperglycemia-induced excitotoxicity and full-blown T3D. It has also been suggested that a pathway of hyperglycemia-induced excitotoxicity is based on the alteration of KATP functions in diabetes. The KATP contains pore-forming units Kir6.1 or Kir6.2 and sulfonylurea (SUR) binding sites (SUR1, SUR2A, or SUR2B). As the Kir6.2 mRNA exists in the brain and overlaps with SUR1 mRNA, it indicates that the Kir6.2/SUR1 is a useful biomarker of KATP activity in the brain. Recent studies have demonstrated that in diabetic conditions, hippocampal Kir6.2 expression is reduced. The authors suggest this may consequently lead to increased neuronal excitability and results in the excitotoxicity process [33]. In turn, Lau and others [34] reported expression changes in genes, which are responsible for glutamate activity in neurotransmission. The authors conducted an experiment with STZ-induced diabetic mice and analyzed gene expression by using the PCR method. After 12 weeks they observed a decrease in the transcript levels of several genes, corresponding with two glutamate transporters, almost all of the NMDA receptor subunits, and all of the kainate receptor subunits. The authors confirmed that diabetes strongly affects the gene expression of NMDA and KA receptor subunits rather than AMPA receptor subunits. It is suggested that diabetes significantly influences the transcriptional expression of genes connected with glutamate neurotransmission based on ligand-gated ion channels, which was observed 3 months after the induction of diabetes. Moreover, the authors confirmed the loss of ganglion cells in diabetic mice, which is explained as a result of a glutamate-induced excitotoxicity effect which is hyperglycemia-dependent.  Figure 1 presents a possible way in which hyperglycemia-induced excitotoxicity leads to T3D development.

On the other hand, an increasing number of reports suggest the important role of advanced glycation end products (AGEs) and the processes of their formation and accumulation, which occur during normal brain aging but are increased in diabetic conditions. An increased expression of the specific receptor for AGEs (RAGE) in neurons and glia from cognitively affected diabetic mice has been confirmed. It is also suggested that the interaction between AGEs and RAGE promotes ROS formation, which may result in calcium-dependent excitotoxicity [35]. While AGEs increase the production of ROS, it is suggested that this consequently stimulates the downstream pathways of NAD+-dependent deacetylase Sirtuin 1 (Sirt1), 78 kDa glucose-regulated protein (GRP78), amyloid precursor protein processing, and A*β* production [36].

## 4. Insulin Resistance of the Brain: the Missing Piece of the puzzle?

It is well known that insulin acts through insulin receptors (IR) and is particularly responsible for regulating glucose uptake and utilization, glycogen synthesis, enzyme phosphorylation or dephosphorylation, and modulation of cellular proliferation [37]. Interestingly, some studies report the role of insulin in memory, learning, and cognition [38, 39]. It is also confirmed that insulin is responsible for the maintenance of synaptic plasticity, differentiation, and stimulation of neurite outgrowth [33]. The first notification about insulin's ability to cross the blood brain barrier was suggested in 1967 by Margolis and Altszuler [40], who experimentally confirmed an increase of insulin concentration in Cerebrospinal fluid (CSF) after peripheral infusions of this hormone. Ten years later, Woods and Porte [41] observed high insulin concentration in plasma after the infusion, but relatively low concentration in CSF, and they confirmed a nonlinear correlation between insulin plasma and CSF concentration. Undeniably, insulin resistance in T2DM, and the corresponding peripheral hyperinsulinemia, reduces insulin transport through the blood brain barrier (BBB). Insulin receptors in the brain and those in peripheral tissues are similar in pharmacological and kinetic properties; however, they are different in molecular size: it is confirmed that *α* subunits of IR of the brain (IR-A) is smaller than *α* subunits of IR of peripheral tissues (IR-B). The brain IR differs also in antigenicity and in the degree of glycosylation. Furthermore, when peripheral IR is downregulated in response to insulin excess, its counterpart in the brain does not record such downregulation [42]. However, peripheral insulin resistance triggers insulin resistance of the brain [43]. More and more recent evidence suggests the pivotal role of insulin resistance and insulin receptor signaling in neurodegeneration, especially in AD [44, 45].

The mechanism of insulin resistance in AD is also related to insulin-like growth factor 1 (IGF-1) resistance, and insulin receptor substrate 1 (IRS-1) disability, probably triggered by A*β* oligomers [13]. In physiological circumstances, insulin connects with the insulin receptor and triggers intrinsic IR tyrosine kinase activity. Activation of IR results in phosphorylation of tyrosine residues of insulin receptor substrates: IRS-1 through IRS-4. This stimulates the signaling pathways of insulin related to phosphoinositide 3-kinase (PI3K) and downstream cellular responses that facilitate synaptic plasticity and memory [44, 46]. The hypothesis based on IGF resistance and the deficiency involved in the pathogenesis of AD seems to be confirmed by the results of the early stages of this pathology, including CSF levels of insulin, IGF-1, nerve growth factor, and glial derived neurotrophic factor levels, which were significantly decreased in contrast to increased neuroinflammatory markers (related to age controls). More importantly, a postmortem diagnosis of AD was confirmed in all patients [47]. The hippocampal formation and cerebellar cortex in Alzheimer's Disease without concomitant diabetes exhibit a significant reduction of responses to an IR→PI3K signaling pathway with an accompanying reduction of responses in the IGF-1R→IRS-2→PI3K signaling pathway [48]. A*β* oligomers promote the additional removal of IRs from the cell surface and redistribution to the body cells. This results in blocking neuronal insulin signaling. Abnormal activation of NMDA receptors by A*β* oligomers leads to calcium influx, neuronal oxidative stress, and impaired synaptic plasticity [44]. Reduced glucose utilization occurs as a result of defects in the insulin receptor signaling pathway. This may lead to a decrease in ATP production and a defect in ion channel activity, and in consequence to excitotoxicity [49]. Insulin resistance of the brain may contribute to AD by A*β* accumulation, tau phosphorylation, oxidative stress, proinflammatory cytokines, AGEs, dyslipidemia, and apoptosis [43]. Other studies demonstrate that hypoinsulinemia may also lead to persistent phosphorylation of tau protein (mice model), a protein particularly connected to AD [50].

Taking into account the world diagnostic standard, it seems important to emphasize the role of insulin homeostasis in annual diagnostics, not only in patients with disorders in the carbohydrate metabolism or with a history of diabetes in the family. The regular control of fasting glucose and insulin, and assessment of insulin resistance (HOMA-IR) as a useful biomarker of prediction of diabetes, might inhibit the escalation of the T2D epidemic.

## 5. Amylin: an Interesting Connection between T2DM and AD

Amylin, also known as islet amyloid polypeptide (IAPP), is a hormone, which is cosecreted with insulin from pancreatic islets *β* cells. Physiologically, after nutrient stimulation, the insulin:amylin molar ratio in peripheral circulation is approximately 10-100:1; however, in diabetic conditions, amylin concentration decreases (type 1 diabetes) or its production is impaired (type 2 diabetes). Amylin, as a neuroendocrine hormone, affects CNS through the specific receptors found in the nucleus accumbens, the dorsal raphe, and the area postrema in the rat brain. It is suggested that the postrema area in brainstem is very sensitive to amylin, and, in addition, this area is deprived of BBB; hence it is available for other peptides [51]. What is more, recent research reports that the amylin may act, via interleukin-6 (IL-6), as a mediator in the neural pathway and stimulate production of endogenous IL-6 in the hypothalamus, which leads to increased leptin sensitivity in the brain and consequently results in a decrease in appetite [52, 53]. The link between the IAPP precursor (proIAPP) and T2D and the loss of *β* islet of Langerhans is interesting. It is suggested that proIAPP facilitates for IAPP the formation and aggregation of amyloid fibrils in the pancreas, which results in amyloid-induced apoptosis of *β*-cells [54]. It is thought that hyperglycemia conditions impair N-terminal processing of proIAPP, and the abnormal form of proIAPP provides granules for IAPP amyloid formation [54]. Taking this into consideration, it seems reasonable to focus on the impairment of proIAPP processing as a new therapeutic target for T2D. Undeniably, these pathological amyloid fibrils are characteristic for patients with T2D; however, it is still not clear whether IAPP amyloid formation is a reason for, or a consequence of T2D [55]. Nevertheless, insulin dependent patients with T2D manifested a higher degree and dominance of IAPP amyloid in comparison to patients without insulin treatment [56]. An increasing number of studies support the existence of IAPP in the human brain; however, instead of insulin, the efficiency of IAPP crossing the BBB is extremely low [57, 58]. Also the IAPP receptors and immunoreactivity in AD brain tissues have been proved [57, 59–61]. More importantly, it is suggested that IAPP is produced in the human brain as the IAPP mRNA has been found in AD brain tissues [59, 62]. In 2014, Ghiwot et al. [63] proposed the hypothesis that the IAPP involved in T2D could cross-seed and enhance A*β* misfolding to aggravate the pathology of AD. The aim of the study was to establish IAPP presence in the brain of nontransgenic murine models and determine the concentration of IAPP in human cerebrospinal fluid in AD and non-AD cases. The authors proved the existence of IAPP in the brain, the association with AD models, and the escalation of A*β* oligomerization in vitro; however, they did not observe a correlation between peripheral levels of IAPP in the blood and AD pathology. Nevertheless, they suggest that possible IAPP resistance may be a novel molecular therapeutic target for type 3 diabetes. Recent studies focus on a potential therapeutic role for *β*-site APP-cleaving enzyme 2 (BACE2), an aspartyl protease, in hyperamylinaemia as a consequence of type 2 diabetes. Rulifson et al. [64] identified the specific structure of the mature human IAPP sequence that is receptive to proteolytic activity initiated by BACE2. The proteolysis affects the modulation of IAPP fibrillation and IAPP protein degradation. The authors have confirmed the beneficial influence of BACE2 on *β* cells, which may be useful as a new therapeutic procedure in T3D.

## 6. Diminished Cerebral Glucose Metabolism as a Useful Indicator of AD Development

Cerebral glucose metabolism includes transport through the BBB, glycolysis, tricarboxylic acid cycle, and ultimately oxidation with carbon dioxide and water production for the full provision of (ATP) and another kind of high-energy source [65]. Most of the glucose provided to the brain via BBB is used to regulate the synaptic transmission and control of neuron resting potential. Similar to the liver or muscle, glucose in the brain is stored as glycogen, particularly in astrocytes; however, its concentration is relatively low. Nevertheless, astrocyte glycogen is a reserve of glucose, and in the case of an accidental hypoglycemia condition it is responsible for maintaining neuronal functions [66]. Some evidence has established that diminished cerebral glucose metabolism (DCGM) is a key point in Alzheimer's Disease [67]. It is confirmed that DCGM appears relatively early in Alzheimer's Disease, is definitely region-specific, and relates to people at genetic risk of AD. The main risk for late-onset AD is the status of apolipoprotein E4 (APOE4) gene mutation [68, 69]. It is said that APOE4 carriers demonstrate a low cerebral glucose metabolic rate, with no comorbid cognitive impairment and increased A*β* plaque accumulation, which makes the DCGM a marker of potential AD which begins several dozen years before cognitive debilitation is confirmed [69]. Interestingly, in DCGM the brain's capacity to utilize glucose as fuel is diminished up to 25% [67]. Modern, noninvasive neuroimaging techniques used to play a very important role in identifying the metabolic biomarkers. The most common approach to analyzing brain metabolism is positron emission tomography (PET), with fluorine-18 (18F)-labeled 2-fluoro-2-deoxy-d-glucose tracer (FDG), which simulates a combination of both glucose transport and subsequent phosphorylation [70]. The most important advantage of FDG is comparable to glucose transport into tissues, and also to the brain, and the ability to be phosphorylated by hexokinase. However, FDG cannot be further metabolized to fructose-6-phosphate, so it remains in the tissue as FDG 6-phosphate. Thereby, FDG uptake is comparable to glucose uptake but without subsequent metabolism towards CO_2_ [71]. DCGM via FDG demonstrates the characteristic pattern, particularly affecting the posterior cingulate, temporal, parietal, and prefrontal regions of the brain [67, 72, 73]. It is suggested that diagnosis performed via FDG-PET in patients with long-lasting T2D will help to find an answer the question whether the DCGM would be a potential marker of type 3 diabetes development.

## 7. Experimental Type 3 Diabetes Treatment

Treating T3D as a neurometabolic disorder gives researchers the opportunity for novel approaches to therapy for AD, including metabolic regulation as a priority. Comorbid metabolic disorders are hyperglycemia, insulin resistance, and IGF-1 resistance, which result in diminished cerebral glucose metabolism. The most recent proposed treatment is based on brain-penetrating peroxisome proliferator-activated receptors (PPAR) *δ*/*γ* agonists, responsible for the remediation of neurocognitive deficits and neuropathology confirmed in AD. Nevertheless, in contrast to the other PPAR-targeted diabetic drugs previously evaluated in AD, T3D-959 is not a thiazolidinedione. Clinical trials conducted in phase 1 have confirmed that T3D-959 is extremely safe, no drug-related adverse events were observed, and no maximum tolerated dose (MTD) was reached [74–76]. The trials performed on STZ-induced diabetic rats included initiated neurodegeneration with increased tau protein, A*β*42, A*β* protein precursor (A*β*PP), ubiquitin, and reduced levels of synaptophysin, IGF-1 receptor, IRS-1, protein kinase B (PKB), p70S6 kinase (p70S6K), mTOR kinase, and phospho-GSK-3 beta S9 (S9-GSK-3*β*). The effect of T3D-959 treatment reduced most of the neuropathological complications and normalized insulin/IGF signaling and abrogate neuroinflammation, thereby it is suggested that T3D-959 may reduce neurodegeneration and cognitive impairment [76]. Current phase 2a trials are based on the FDG-PET imaging measurement of the cerebral metabolic rate of glucose, and the evaluation of changes in hippocampal functional connectivity, cognitive functioning, and metabolome, during varying repeat doses of T3D-959. This is the first exposure for this treatment in patients with AD. The authors emphasize that the results support future clinical testing of T3D-959 in a larger, phase 2b, randomized, controlled trial [75]. It is definitely a novel and supported neurometabolic approach in AD treatment, which may turn out to be more effective in patients with T3D.

## 8. Conclusions

It is indicated that T3D may be the result of an overload of calcium brain fluid concentration connected with hyperkalemia, which suggests that special attention should be paid to the contribution of parathormone and calcitonin in the regulation of calcium levels in patients with diabetes. According to worldwide diabetes standards, fasting insulin concentration and HOMA-IR assessment matter in AD and should be included to prevent insulin resistance development connected with T2D.

As has been confirmed, the IAPP plays a crucial role in the formation and aggregation of amyloid fibrils in pancreatic *β*-cells, and enzymatic modulation of IAPP fibrillation seems to be a new therapeutic target for T3D. Furthermore, diminished Cerebral Glucose Metabolism is proposed as a useful marker to predict AD, especially in APOE4 carriers, many years before cognitive impairment is confirmed, and taking into consideration research on DCGM in T2D is suggested, since this would be a unique test for T3D development.

Finally, T3D-959 clinical trials (phases 1 and 2) confirmed its safety and the remediation of neuroinflammatory and neurodegeneration biomarkers, and it seems as if treating AD as a neurometabolic disorder, resulting from hyperglycemia and insulin signaling pathway disturbances, would uncover attractive new approaches in the treatment of T3D.
